# Leadership assumptions on implementation of patient involvement methods

**DOI:** 10.1186/s12913-021-06497-y

**Published:** 2021-05-26

**Authors:** Kathrine Håland Jeppesen, Kirsten Frederiksen, Marianne Johansson Joergensen, Kirsten Beedholm

**Affiliations:** 1 Department of Public Health, Research Unit for Nursing and Health Care, Aarhus University, Bartholins Allé 2, 8000 Aarhus C, Denmark; 2 Department of Clinical Medicine, Horsens Regional Hospital, Sundvej 30, 8700 Horsens, Denmark

**Keywords:** Patient involvement, Implementation, Leadership, Organisational changes, Organisational learning

## Abstract

**Background:**

From 2014 to 17, a large-scale project, ‘The User-involving Hospital’, was implemented at a Danish university hospital. Research highlights leadership as crucial for the outcome of change processes in general and for implementation processes in particular. According to the theory on organizational learning by Agyris and Schön, successful change requires organizational learning. Argyris and Schön consider that the assumptions of involved participants play an important role in organizational learning and processes. The purpose was to explore leaders’ assumptions concerning implementation of patient involvement methods in a hospital setting.

**Methods:**

Qualitative explorative interview study with the six top leaders in the implementation project. The semi-structured interviews were conducted and analyzed in accordance with Kvale and Brinkmanns’ seven stages of interview research.

**Result:**

The main leadership assumptions on what is needed in the implementation process are in line with the perceived elements in organizational learning according to the theory of Argyris and Schön. Hence, they argued that implementation of patient involvement requires a culture change among health care professionals. Two aspects on how to obtain success in the implementation process were identified based on leadership assumptions: “The health care professionals’ roles in the implementation process” and “The leaders’ own roles in the implementation process”.

**Conclusion:**

The top leaders considered implementation of patient involvement a change process that necessitates a change in culture with health care professionals as crucial actors. Furthermore, the top leaders considered themselves important facilitators of this implementation process.

**Supplementary Information:**

The online version contains supplementary material available at 10.1186/s12913-021-06497-y.

## Background

Implementation of patient involvement initiatives is high on government agendas in Western societies [19] and considered a health care changing process [17]. Existing research highlights the importance of leadership for the outcome of change processes in general and for implementation processes in particular [7, 8, 10, 20]. Thus, in this study, our starting point was that knowledge of leadership is important in an implementation process, and we aimed to explore what the leaders found significant in their leadership and how they acted accordingly.

**Fig. 1:**
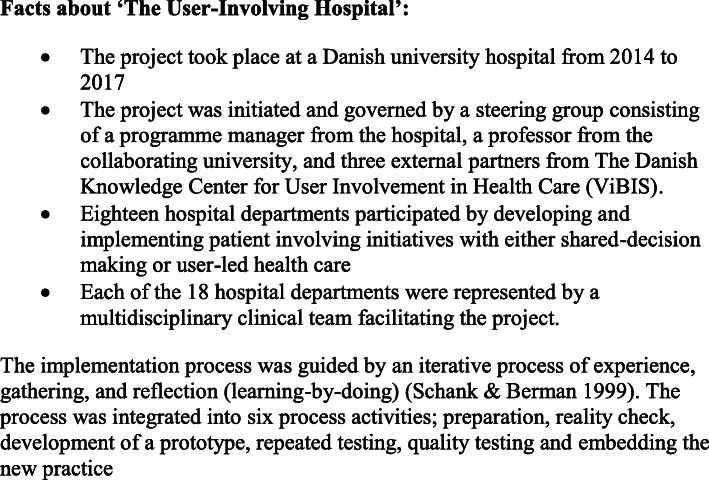
Facts about ‘The User-Involving Hospital’

Our study was conducted among a group of top leaders concerning a large-scale implementation project at a Danish university hospital called ‘The User-involving Hospital’ [9] (Fig. 1). As a part of this project, 18 hospital departments implemented patient-involving initiatives in the form of either shared-decision making or user-led health care. Our study focused on leadership aspects in this implementation process and relied on the theory of Argyris and Schön of organizational learning [1]. According to Argyris and Schön [1], organizations only change if organizational learning takes place. In line with Argyris and Schön, the top leaders considered the implementation of patient involvement a change process that necessitates a change in culture with health care professionals as crucial actors. Furthermore, the top leaders considered themselves as important facilitators of this implementation process.

### Implementation and the role of leadership

Several studies suggest the importance of leadership in the successful implementation of sustainable change [6, 16]. Winther and Nielsen [20] highlighted the interaction between top management and employees as essential in the transformation of implementation processes. Another study [8] pointed out the top management’s ability to communicate and convince employees of the need to change as a key factor in successful implementation of new initiatives. Furthermore, top management support and commitment to change play a decisive role. Kotter [10] demonstrated the challenges in rooting new behaviors and social norms in an organization when the pressure and need for change fade. Kotter argued [10] that changes must become an embedded part of the organization. A study by Chreim et al. [6] concluded that persistent leadership is necessary for a successful healthcare change and indicated that a division of the components in a change process as well as finding actors to lead the change process is an important strategy to success. Furthermore, Scott et al. [16] argued that a behaviorist rewarding approach such as transactional leadership is insufficient. However, integration of transactional and transformational leadership is necessary to inspire and sustain change.

### Implementation of patient involvement

According to Lord and Gale [12], patient-involving initiatives can be difficult to implement because of professional roles, values and changes in power structures. In line with this, research suggests that patient involvement affects the interpersonal relation between the health care professional and the patient [4] by changing existing power structures and introducing a more partnership-oriented approach. For health care professionals to be able to adopt the new roles required by patient-involving initiatives, existing organizational structures and cultures must be changed. Ponte et al. [14] pointed at the need to develop a shared understanding of the meaning and goal when implementing patient involvement in organizations as a part of the leadership responsibility. A study by Luxford et al. [13] considered strong committed senior leadership as essential in the change process of patient involvement. A main barrier in changing the organizational culture is the length of the transition to shift more focus to the patient [13]. Coulter [5] pointed to engagement and effective leadership at all organizational levels from top management to health care professionals, empowering the change process in the organization. Furthermore, clear goals in the implementation process are highly important: *“Clear goals and effective methods for communicating these at every level, from board to management to frontline workers to patients and families, are essential for spreading and reinforcing patient-centred values and procedures”* ([5] p. 14).

Thus, this study focused on the top leaders’ role as decision-makers and responsible for the implementation of patient involvement. The study was inspired by the theoretical framework of Argyris and Schön, [1] on organizational learning. Argyris and Schön suggest that organizational learning can take place as either single- or double-loop learning. Single-loop learning takes place with corrective actions permitting the organization to continue current policies or achieve current objectives. Double-loop learning takes place when new actions involve modification of the organization’s underlying norms, policies and objectives. Hence, double-loop learning is necessary to achieve sustainable change [1]. Thus, we consider the implementation of patient involvement methods a process calling for organizational learning.

Argyris and Schön use the concept ‘theory of action’ to describe how actions are guided by what can be understood as tacit structures. In our study, ‘theory of action’ is conceptualized as assumptions related to the implementation process. Assumptions cover both espoused theory and theory in use. Actors express their espoused theory in the form of meaning, values, and beliefs, while theory in use concerns the actual behavior in situations, which are not necessarily in accordance with the actor’s espoused theory:

*“When someone is asked how he would behave under certain circumstances, the answer he usually gives is his espoused theory of action for that situation. This is the theory of action to which he gives allegiance, and which, upon request, he communicates to others. However, the theory that actually governs his actions is this theory-in-use.” (Argyris and Schön* 3*: 6–7).*

In this study, we explored leadership assumptions concerning implementation of patient involvement in a hospital setting by asking what the leaders found important in their leadership and how they acted accordingly. In this way, we focused on the leaders’ espoused theories in relation to the process of the implementation project ‘The User-involving Hospital’. Our study is a part of a more comprehensive study on leadership and implementation of patient involvement.

## Methods

The study was partly based on our preunderstanding retrieved from literature on the importance of leadership during implementation of patient involvement [6, 16] and partly on the theory of Argyris and Schön on institutional learning. This led us to the assumption that the leaders of The User-involving Hospital” would be crucial for the implementation process. Thus, we wanted to explore what the leaders found important in their leadership and how they acted accordingly. The study is therefore designed as a qualitative explorative interview study involving individual interviews with top leaders of the hospital and members of the implementation project steering group.

### Informants

All members of the steering group together with the hospital leaders were invited by email to participate in the study; seven informants accepted the invitation. The sampling strategy was purposive [18] to gain insight into what the leaders as decision-makers assumed to be important in their leadership during the implementation process. All informants were included because they were all decision-makers in the implementation project. The researchers had a professional relationship to some of the participants from previous collaboration on research and patient involvement. Unfortunately, one of the interviews was cancelled due to serious illness and death. Due to anonymity, we refrain from elaborating further on the background of the informants.

### Data collection

The interviews were guided by a semi-structured interview guide. The guide was developed by the first author in close collaboration between the co-authors experienced in qualitative research to ensure coherence between the themes of the interview guide and the purpose of the study. The themes of the interview guide focused on the role, thoughts and expectations of the decision-makers, the change process, co-operation and levels of management. The questions were mainly formulated as open-ended questions such as “What happened in this phase?” to let the informants freely express both explicit and implicit assumptions. Furthermore, a timeline on the implementation of events was used to probe the informants’ recall of their thoughts and expectations during the implementation process and to make them reflect on certain events. Throughout the interview phase, the interview guide was assessed and revised in step with the interview group’s increased pre-understanding. All interviews were conducted by the first author. They took place as face-to-face interviews at the office of the informants or in a meeting room; the interviews lasted approximately 1 h. All informants received written and oral information on the project aim and were informed that findings of this study would contribute to important knowledge on the implementation process. Informed consent was signed by all informants. All interviews were digitally recorded and transcribed verbatim by the first author in accordance with Kvale and Brinkmann [11]. Besides from the audio recording, the interviewer took notes during the interview.

### Data analysis

The coding process was conducted by the first author. Coding strategy and data interpretation were discussed among the authors in an ongoing process. The informants were strategic chosen. All leaders from the steering group were invited into the study, data saturation could not be a goal.

The authors discussed the number of participants in the study based on purposive sampling. Data saturation was discussed in line with the explorative and hermeneutical approach in the study.

The data analysis process included two phases: First, the analysis focused on the informant’s self-understanding [11] aiming to identify assumptions related to different aspects of the implementation of patient involvement. During the coding process, patterns emerged on the overall assumptions about the desired goal of implementing patient involvement and on how to obtain project success. In this first analytical phase, a hermeneutical approach was used to determine meaning structures in the interviews [11], and categorize assumptions. In the second phase, Argyris and Schön’s theory on organizational learning was applied as a theoretical frame for meaning interpretation. In this phase, the analysis moved from informants’ own words and self-understanding to what Kvale and Brinkmann denote as critical common sense and theoretical understanding.

## Results

The leaders shared an overall assumption about how to achieve the goal of implementing ‘The User-Involving Hospital’. Two aspects were assumed to be crucial to obtain success: i) “Assumptions on the changes needed among health care professionals and their role” and ii) “Assumptions on the leaders’ own role in the implementation process” (Table 1). As no major differences were identified in the assumptions between informants, all meaning units were merged into shared assumptions.

**Table 1:** Schematic overview over leaders’ assumptions

**Leaders’ overall assumption**	
Implementation of ‘shared decision making’ and ‘user led care’ has the potential to become a culture-changing project and thereby prepare the ground for large-scale implementation of patient involvement.	
**Assumptions on how to obtain success**	
The health care professionals’ role in the implementation process requires:	The leaders’ own role in the implementation process is crucial:
• Change in mindset	• Management attention is necessary for culture change to take place
• Allocation of time to allow health care professionals to perform the changes	• Management must facilitate meaningful projects

### Leaders’ overall assumption – a culture-changing project

The overall common assumption was that culture change is decisive for success. This was expressed by one of the leaders:

“*It has to be culture-changing, not just a project. We want to call it a movement not just a project as projects tend to come to an end. Movements create changes, new ways of working. Therefore, a criterion for success will be that these initiatives will continue and spread leading to a new culture and new ways of collaborating with patients.”*

Hence, the vision is that the patient-involving culture will spread throughout the organization leading to change. Several of the leaders assumed that the project would create a setting for a culture change through the implementation of patient involvement methods. Hence, the assumption that a change in culture is a precondition for the project to be successful involves what Argyris and Schön describe as double loop learning leading to a permanent culture change in an organization.

Another informant reflected on the potential of the project: *“We have 21-22 projects ( …*) *and it is really a handful [of projects], but I thought it could be very exciting to see if you can create change by implementing a large-scale project ( …*)” . This reflection was put forward in connection with considerations on the number of hospital departments participating in the implementation project. Later the informant added that they would both succeed and not succeed, because not every hospital department would be able to develop meaningful methods.

When asked about what characterizes the projects, some leaders emphasized the possibility of developing a project where they could be first-movers on delivering how-to-do results when working with patient involvement: *“It has been a first-mover project to show how this can be done”*. Furthermore, one informant elaborated: “*Our goal was to develop methods to make it possible for departments all over the country to find methods to increase user-involvement in their department”*. This expresses a concrete vision of the project to develop methods to involve patients and to spread these visions to other hospitals. This visionary assumption was highlighted when the informant reflected on expectations to the project, as the informant expressed it was an overall aim to create local results and be influential in a national context.

### How to obtain success – assumptions of the health care professionals’ role in the implementation process

The leaders shared the assumption that health care professionals are crucial to successful implementation of patient involvement and culture change. This was expressed as a need for health care professionals to change not only their roles but also their mindset. Furthermore, the leaders also mentioned that time necessary for the health care professionals to create meaningful projects.

#### A change in mindset

The informants considered the health care professionals as essential actors in the culture-changing process involving a change in the health care professionals’ work habits and behavior.

One informant assumed that it was possible to develop a patient-involving project that would push and change the culture across the entire hospital, both at top management level and at clinical departments. In relation to the changes among health care professionals an informant said:

*[ … ] It might seem small, but it is a huge revolution to change the mindset concerning the ways patients are met. It is not easily done disconnected to the context you are a part of, the institution you represent and is embedded in”*

This indicates that the project might affect the mindset and daily work of health care professionals. The informant assumes that what seemed to be a small project has a radical influence on both the patient perspective and the work habits and culture of health care professionals.

Another informant was concerned about the willingness of hospital departments to participate in the project, which the informant believed depended on their understanding of the roles of nurses and doctors. This willingness was expected to be triggered by the demand to implement either shared decision-making or user-led care. Some of the informants explained that they found that the mindset of doctors differed from that of nurses; the main priority of doctors is treatment whereas nurses value the inclusion of the patient’s perspective;

*“There is just more interest in patient involvement among nurses. It is as simple as that. Maybe it is due to the professional attitude among nurses while doctors with responsibility for the treatment administer a very specialized but limited area.”*

Another informant wondered: *“But can this project be a platform to really push the culture, or will projects just be scattered randomly? …*.” The informant expressed a concern that the projects on the patient involvement methods would develop differently than intended. Thus, concerns were expressed that the culture to be changed is so rooted in the health care professionals that change would be difficult.

#### Allocating time is necessary

Time was considered a precondition for developing new patient-involving methods, and informants also assumed that it was possible for health care professionals to involve patients provided the necessary time was allocated. Some leaders drew attention to time as a barrier in creating a platform for a culture change. One of the informants explained: *“I believe the hardest part is to find time and room [for the project] because it takes a long time, especially in the beginning when you plan and establish the project; to get it started and make local descriptions and persuade any sceptics in the department”*.

The informant highlighted time as a key factor in the beginning of the project to allow the staff to create and develop their own projects. The informant expressed that time could be an obstacle, because the project requires that time is allocated. Furthermore, another obstacle was the need to pay attention to the critics and a need for initiatives to get them onboard. Another informant also highlighted the importance of time and elaborated: “*( …) and deep down maybe they [the health care professionals] don’t need so many presentations or descriptions, I mean they may need a few for inspiration, but otherwise they need the time themselves to think and talk things over”.* Thus, the informant believed that creating good working conditions for the staff was maybe more important than providing illustrative examples of the projects.

### How to obtain success - assumptions on the leader’s own role in the implementation process

The leaders agreed that there was a need for management attention to the implementation process. Furthermore, they expressed that an important prerequisite for the success of the entire project was that the project was perceived as meaningful for the health care professionals.

#### Management attention

Assumptions that leadership is important during the process was expressed when the informants were asked about key events during the implementation process. All informants mentioned the so-called top management visits, where the steering group and top management visited the hospital departments and each project group presented their patient involvement project receiving feedback and acknowledgement. One informant expressed:

*“I think it’s been very important to have a visit from the top management. We actually hear that it means something, that the top management is interested and comes to visit.”*

Another informant had a similar assumption:

*“I think the visits from the top management have been rather important [ … ], because it’s my understanding that the departments have felt it was important what they had done [ … ]”.*

The attention from the management was considered an acknowledgement of both the projects and the hospital departments.

One informant explained how the experience of the visits had been important to him as a leader, but he also highlighted the health care professionals and the hospital departments. In this way the informant demonstrated an awareness of the importance of the health care professionals’ own contribution to the project.

Another informant explained the experience of the visits from a leader’s point of view: *“They [members of the project groups] are proud of what they do, all of them actually ( …*). *They also tell that they change more than what their little project indicates”.* The informant focused on the importance of the health care professionals’ own contribution to the project and highlighted the health care professionals’ experiences of contributing to the entire project and stimulating the culture-changing process.

Thus, leaders expressed that leadership is important in creating the culture change. Two of the informants claimed that culture change is a result of management pressure. One of them said: “*[ …*] *I believe the culture will emerge now because of the pressure from the management and the fact that something happens in the departments”.* The informant also expressed an expectation that one process at a hospital ward related to a specific procedure and group of patients would spread as a culture movement within the departments and outside to other departments at the hospital.

#### To facilitate meaningful projects

When asked about important matters during the implementation process, one informant explained:” *Well, management at all levels must be in place; this is the best. And it has to make sense professionally”*. This statement illustrates another view on creating culture change - that the project has to be perceived as meaningful for health care professionals. The informant elaborated on the point that the projects had to make sense for the health care professional on a short-term basis. Another informant highlighted the patient involving methods as a way of creating a setting leading to meaningful projects. This view required the leaders to ensure that tasks were meaningful to the health care professionals. The leaders assumed that a crucial tool in this process was that the project groups themselves formulated and developed the patient involvement project choosing either shared-decision making or patient-centered care as a method.

To sum up, our analysis indicated that the top leaders shared the assumption that successful implementation of patient involvement methods, a culture change had to take place and that efforts from both health care professionals and management were necessary. Moreover, leaders agreed that health care professionals are key actors in the implementation process, which requires changes in mindset and work routines. The leaders’ own role was to ensure management attention and direction to facilitate the necessary changes. This implied that the leaders did not assume that patient involvement could be implemented as a top-down project but should be carried out in close collaboration with the health care professionals, who should have considerable influence on the specific projects in their departments.

### Theoretical interpretation

The leaders described that both single- and double-loop learning was necessary for the implementation of patient involvement methods to change not only staff work routines, but also habits and behaviors. Hence, the leaders’ espoused theory showed that implementation of patient involvement methods requires a change in the health care professionals’ theory in use.

The leaders acknowledged the importance of leadership and highlighted their own role as crucial during the process to create the necessary foundation for organizational change. In this way, leaders pointed to their own responsibility as facilitators of the culture change process. Hence, the leaders did not understand the change process as a top down process.

The analysis showed a general notion of culture as central and changeable, and an understanding that the process of change has to spread both horizontally and vertically. According to Argyris and Schön [2], an element of organizational learning is:

*“An overarching sense of organizational learning that refers broadly to an organization’s acquisition of understandings, know-how, techniques, and practices of any kind and by whatever means. In this sense, organizational learning is pervasive and, in itself, neither good nor bad”* ([2], p. 21).

Organizational changes do not only involve behavioral change for those involved. The leaders’ espoused theory illustrated an awareness of this realizing that patient-involving initiatives imply a culture change and more radical changes within the organization.

Based on this understanding, many challenges in the process of patient involvement could be associated challenges in organizational learning processes. One of these challenges could involve the behavior of those involved in the implementation process. Argyris and Schön [2] argued that it is important to distinguish between starting to see things in new ways and beginning to act on the basis of insight as an institutional agent ([2], p. 23). Thereby, they acknowledged that fit was important to health care professionals to work with self-defined patient involvement visions. To health care professionals, it was necessary that the visions were meaningful and made professional sense for them to act on the visions.

## Discussion

Based on the theoretical framework of Argyris and Schön, we suggest that leaders are aware of the challenges in successful implementation of the vision of “The user-involving Hospital” and that they consider health professionals and the organization as capable of creating a foundation for a meaningful culture change. Based on this understanding it could be argued that a culture change involves a cognitive change among health care professionals. The leaders’ assumptions concerning the health care professionals pointed to a need for changes in behavior and habits in connection with specific patient involvement methods. This might be challenging since a learning process involving behavioral change and change of habits takes place in a specific and complex organizational structure. Luxford et al. [13] pointed to the length of time as a barrier to an increased patient-centered focus. Thus, changes might be more complex when involving processes of changing theory in use among health care professionals. Research shows that challenges in rooting new behaviors and social norms point at the importance of it becoming an embedded part of the organization [10].

Existing research on leadership in the implementation of patient involvement shows that leadership focus on all levels is important [5]. Thus, the findings of this study are not unique. However, this study provides insight into the leaders’ assumptions of what is needed to facilitate an implementation process. Their assumptions support the theory of Argyris and Schön on organizational learning and point to the leaders’ acknowledgement of the need for them to facilitate both single- and double-loop learning processes that will cause change in the theory in use among health care professionals.

### Limitations

The aim of the study was to explore leaders’ assumptions concerning the implementation of patient involvement methods in clinical practice. The informants were all members of the steering group of the implementation project together with the hospital director. The informants provided in-depth information on their assumptions regarding the implementation of the project ‘The User-involving Hospital’. Thus, it was not relevant to expand the group of informants. We found it important to address the whole steering group due to our assumption that this group had crucial influence on the implementation process. However, in future, it would be interesting to conduct studies among leaders from other hospitals or implementation projects to explore and compare more aspects of leadership and their association with success in implementation of patient involvement projects.

The study was based on interviews providing in-depth insight into the explicit considerations and experiences of leaders, which in our study was conceptualized as leadership assumptions.

The theoretical analytical perspective by Argyris & Schön was useful in exploring the leadership assumptions in organizational change processes. However, interviews do not uncover the dynamics and possible incoherence between the leaders’ espoused theory and theory in use. This study offers insight into the leaders’ assumptions focusing on the espoused theory in relation to both single- and double-loop learning.

## Conclusion

The study demonstrated that the hospital top leaders shared some basic assumptions on the implementation of patient involvement methods. Based on the theory of Argyris and Schön, this study suggests that the leaders’ espoused theory demonstrated an understanding that implementation of patient involvement methods requires a culture change. The leaders acknowledged the health care professionals as key actors of this cultural change process and were aware that the projects should be perceived as meaningful to health care professionals to be successful. Moreover, the leaders acknowledged the importance of leadership and their own role during the process and highlighted this as crucial to create the necessary conditions for the health care professionals to facilitate change.

## Supplementary Information

**Additional file 1.** Interview guide: Central leaders and their assumptions on the implementation process of the User-led Hospital project.

## Data Availability

The datasets during the current study available from the corresponding author on reasonable request

## Abbreviations

KHJ: Kathrine Håland Jeppesen
MJJ: Marianne Johansson Joergensen
KF: Kirsten Frederiksen
KB: Kirsten Beedholm
