# Supplementary material for: Leadership assumptions on implementation of patient involvement methods
Source: BMC Health Serv Res. 2021 May 26;21:505. doi: 10.1186/s12913-021-06497-y (PMC8152124; doi:10.1186/s12913-021-06497-y)
Supplement: Supplementary file 1 — Additional file 1. Interview guide: Central leaders and their assumptions on the implementation process of the User-led Hospital project. [file 12913_2021_6497_MOESM1_ESM.docx]

**Interview guide: Central leaders and their assumptions on the implementation process of the User-led Hospital project**

| **Interview questions** |
| --- |
| Question 1  How did the idea of the implementation project* occur?  * Below referred to as "the project" |
| Question 2  Where does the project derive from, and who is driving force/primary force? |
| Question 3  How would you describe the project? |
| Question 4  How did you get involved in the project?  Why did you agree to be involved? |
| Question 5  What were your thoughts on the project, when you got involved? |
| Question 6  What were your expectations to and reservations about the project? |
| Question 7  How did the project evolve over time? Have there been any particularly important key events? |
| Question 8  The interviewer presents a timeline for the process – and asks questions referring to the timeline: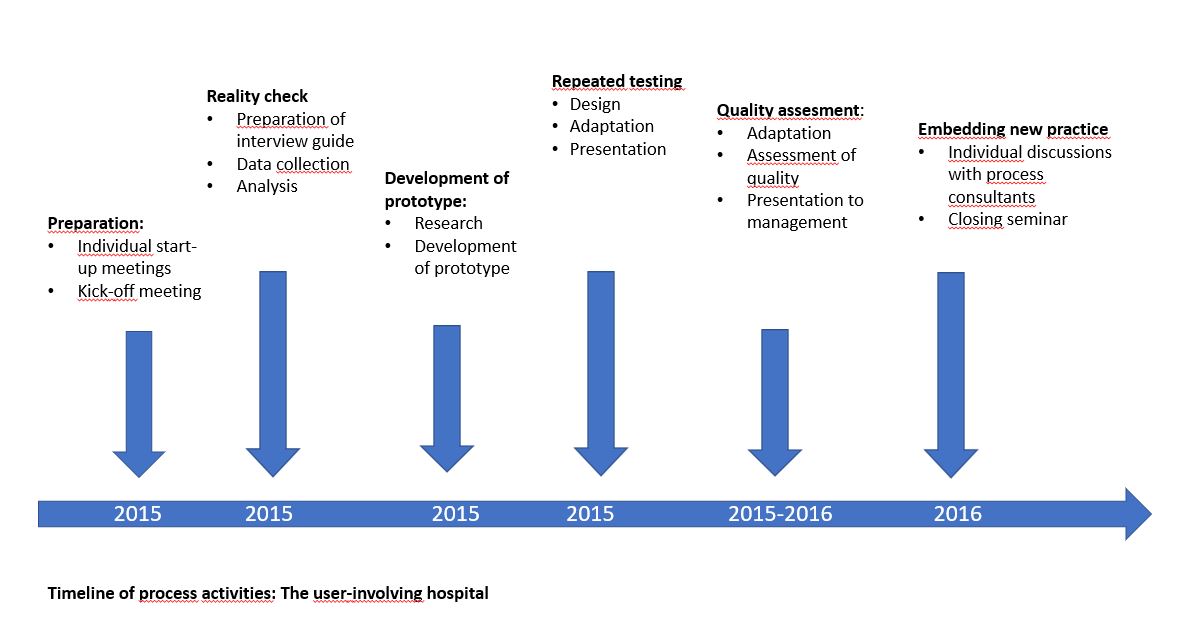  Start – middle - end  What happened in this phase?  Which considerations did you have regarding your support in the project during this this phase?  What did you find easy? What did you find difficult? |
| Question 9  What do you consider to be essential in order to succeed? |
| Question 10  How do you expect the hospital to look like when the project has been completed? |
